# Cerebrovascular disease in patients with antiphospholipid antibody syndrome: a transcranial Doppler and magnetic resonance imaging study

**DOI:** 10.1093/rap/rkae060

**Published:** 2024-05-07

**Authors:** Irapuá Ferreira Ricarte, Lívia Almeida Dutra, Daniela Laranja Gomes Rodrigues, Orlando Graziani Povoas Barsottini, Alexandre Wagner de Souza, Henrique Carrete, Ana Paula Scalzaretto Massaud, Danieli Andrade, Cristóvão Luís Pitangueira Mangueira, Gisele Sampaio Silva

**Affiliations:** Department of Neurology and Neurosurgery, Universidade Federal de São Paulo, São Paulo, Brazil; Department of Neurology and Neurosurgery, Universidade Federal de São Paulo, São Paulo, Brazil; Faculdade Israelita de Ciências da Saúde Albert Einstein, São Paulo, Brazil; Department of Neurology and Neurosurgery, Universidade Federal de São Paulo, São Paulo, Brazil; Department of Neurology and Neurosurgery, Universidade Federal de São Paulo, São Paulo, Brazil; Rheumatology Division, Department of Internal Medicine, Universidade Federal de São Paulo, São Paulo, Brazil; Department of Radiology, Universidade Federal de São Paulo, São Paulo, Brazil; Department of Radiology, Universidade Federal de São Paulo, São Paulo, Brazil; Rheumatology Division, Department of Internal Medicine, Faculdade de Medicina da Universidade de São Paulo, São Paulo, Brazil; Hospital Israelita Albert Einstein, São Paulo, Brazil; Department of Neurology and Neurosurgery, Universidade Federal de São Paulo, São Paulo, Brazil; Hospital Israelita Albert Einstein, São Paulo, Brazil

**Keywords:** antiphospholipid syndrome, transcranial Doppler, microembolic signal, stroke, magnetic resonance, intracranial stenosis, right–left shunt

## Abstract

**Objective:**

Transcranial Doppler (TCD) and brain MRI may be useful in evaluating patients with APS, helping to stratify the risk of cerebrovascular ischaemic events in this population. This study aimed to assess the frequency of brain MRI abnormalities in patients with primary antiphospholipid syndrome, secondary antiphospholipid syndrome and SLE and correlate to TCD findings.

**Methods:**

The study, conducted over four years at two autoimmune disease referral centres, included 22 primary antiphospholipid syndrome patients, 24 secondary antiphospholipid syndrome patients, 27 SLE patients without APS and 21 healthy controls. All participants underwent TCD to assess cerebral haemodynamics, detect microembolic signals and evaluate right-to-left shunts, followed by brain MRI and magnetic resonance angiography. MRI scans were reviewed for acute microembolism, localized cortical infarctions, border infarctions, lacunar infarctions, ischaemic lesions, white matter hyperintensity, micro and macro haemorrhages and arterial stenosis ≥50% of the cervical carotid artery, by two neuroradiologists blinded to the clinical data.

**Results:**

Brain MRI findings were similar between the groups, except for lacunar infarction, more frequent in patients with secondary antiphospholipid syndrome (*P* = 0.022). Patients with intracranial stenosis detected by TCD had a higher frequency of territorial infarction (40% vs 7.5%, *P* = 0.02), lacunar (40% vs 11.3%, *P* = 0.075) and border zone infarcts (20% vs 1.9%, *P* = 0.034).

**Conclusions:**

Patients with intracranial stenosis presented a higher frequency of territorial, lacunar and border zone infarcts, suggesting that evaluating the intracranial vasculature should not be neglected in patients with APS and stroke.

Key messages

- Intracranial stenosis was associated with ischaemic brain lesions in patients with APS or SLE, revealing a potential mechanism for cerebrovascular events.
- Evaluating intracranial vessels in SLE or APS patients with cerebrovascular ischaemic events is crucial.
- Integrating clinical, sonographic and neuroimaging data is essential for comprehensive APS-SLE cerebrovascular understanding

## Introduction

APS is a systemic autoimmune disorder characterized by thrombosis and pregnancy morbidity in the presence of aPL [1–3]. Neurological involvement is common in APS and its occurrence increases morbidity and mortality [4–7]. Ischaemic stroke is the most common and severe arterial complication of APS and one of the leading causes of mortality in this population, making the development of diagnostic tools capable of identifying patients with APS at high risk of developing stroke of utmost importance [8].

The stroke mechanism in APS patients can involve both embolic and thrombotic processes [9]. Small artery involvement leads to lacunar and subcortical ischaemic strokes, while large artery occlusion or stenosis occurs in up to 50% of cases studied through digital angiography [10, 11]. *In situ*, thrombosis is the primary cause of acute occlusion without a cardiac embolic source [12]. Vascular endothelial dysfunction mediated by aPL plays a crucial role in occlusive vasculopathy, involving mechanisms like intimal hyperplasia, accelerated atherosclerosis and microvascular dysfunction [13]. Additionally, APS may also affect extracranial large vessels, such as the common carotid artery (CCA) or internal carotid artery (ICA), with changes like stenosis or occlusion observed in many APS patients with stroke [12].

Among the diagnostic modalities, transcranial Doppler (TCD) and MRI of the brain have proven valuable in assessing neurovascular complications in APS patients. TCD, being non-invasive, enables assessment of cerebral haemodynamics, detecting microembolic signals (MES) and right-to-left circulation shunt (RLS). These parameters help identify APS patients at higher risk of neurological complications, including ischaemic stroke [14–16].

This study aimed to determine the frequency of brain MRI abnormalities in patients with primary antiphospholipid syndrome (PAPS), secondary antiphospholipid syndrome (SAPS) and SLE and relate these abnormalities to TCD findings. Our objective was to identify potential aetiological mechanisms for cerebrovascular ischaemic events in this population and emphasize the importance of evaluating intracranial vessels in patients with APS and SLE.

## Patients and methods

### Patients

Our cross-sectional study prospectively and sequentially recruited adults with primary antiphospholipid syndrome (PAPS), secondary antiphospholipid syndrome (SAPS) and systemic lupus erythematosus (SLE). Enrolment occurred at two leading autoimmune disease referral centres affiliated with renowned quaternary academic hospitals from February 2014 to January 2018.

We included 22 patients with PAPS, 24 with SAPS and 27 with SLE. All patients with SLE met the updated classification criteria for SLE set by the American College of Rheumatology, while all APS patients fulfilled the Sidney criteria [2]. We included a control group of 21 healthy volunteers matched for age and gender with the PAPS group. The exclusion criteria were being under 18 years old, absence of temporal bone window for TCD examination and acute disease activity or acute thrombotic event. Patients who did not undergo brain MRI were excluded from the neuroimaging analyses and patients without peripheral venous access were excluded from the analysis of RLS. The local ethics committee approved this study and written consent was obtained from all participants before the investigation. This study is registered and was approved by the Institutional Review Board (IRB) of the Universidade Federal de São Paulo (unifesp/epm) IN 10/22/2014, with the number 32393714.4.0000.5505.

Consent and clinical data were collected from patients and controls. We assessed epidemiological data, clinical and neurological manifestations of the disease and the presence of traditional cardiovascular risk factors (arterial hypertension, diabetes, dyslipidaemia, chronic kidney disease, obesity, smoking, previous myocardial infarction and presence of carotid stenosis >50%). Echocardiography data were obtained from medical charts. Ongoing antiplatelet or anticoagulation therapy and other relevant medications were also recorded. We reviewed antibody profiles from medical charts during the consultation, which included aPL such as anticardiolipin (aCL) and anti-beta2 glycoprotein I (anti-B2GP1) measured by standardized enzyme-linked immunosorbent assay kits and lupus anticoagulant (by kaolin clotting time and diluted Russel viper venom test). Antibodies were deemed positive when present in moderate or high titres (values above 40 GPL/MPL units for IgG or IgM aCL and above 99th percentile for IgG and/or IgM anti-B2GP1) on two or more occasions at least 12 weeks apart.

Immediately after the initial consultation, the TCD exam was performed. On the day of the TCD examination, we performed complete blood count and coagulation tests (prothrombin time and activated partial thromboplastin time [PT and aPTT]). The patients were evaluated with brain MRI after the TCD exam.

### Transcranial Doppler

The exams were performed by a single certified neurosonologist using a 2 MHz pulsed-wave transducer (DWL, Doppler-Box, Singen, Germany) in all patients and controls through the orbitary, transtemporal and occipital bone windows. Measurements of mean flow velocity (MFV) and pulsatility index (PI) were obtained from the middle cerebral arteries (MCAs), anterior cerebral arteries, posterior cerebral arteries, carotid siphon, intracranial vertebral arteries and basilar artery. The Outcomes and Neuroimaging of Intracranial Atherosclerosis criteria were used to identify 50% stenosis in the intracranial arteries, according to MFV [14, 17].

Cerebral vasomotor reactivity (VMR) was quantified using the breath-holding index (BHI), which measures changes in the MFV of both MCAs induced by breath-holding. BHI was calculated by dividing the percentage increase in mean flow velocity occurring during breath holding by the time subjects held their breath after a normal inspiration (BHI = [MFV at apnoea—MFV at rest]/MFV at rest × 100/30) [18].

All participants were monitored for 60 min in each MCA for MES evaluation. Two 2-MHz pulsed Doppler transducers were fixed using a head frame and the main stems of both MCAs were insonated through the temporal window at a depth of 50–65 mm to capture a small sample volume of 10 mm in length with M-mode. The audible transcranial Doppler (TCD) output was recorded and uncertain cases were resolved by consensus with another certified neurosonologist. The detection and confirmation of MES were performed according to the criteria of the International Consensus Group on Microembolus Detection [18, 19].

Right-to-left shunt (RLS) was investigated using TCD and contrast agents according to the standardized International Consensus Protocol. The test was performed with the patient lying down in a supine position, at rest and after the Valsalva manoeuvre initiated 5 s after agitated saline injection for at least 10 s and MCA monitoring was continued for 25 s. A four-level categorization according to the microbubble (MB) count was applied to quantify RLS [1]: 0MB (negative result) [2], 1–10 MB [3],>10MB and no curtain and [4] curtain. (‘Curtain’ refers to a shower of MBs where a single bubble cannot be identified) [20].

### MRI

Brain MRI scans were acquired using a 3.0 Tesla magnetic field machine (Philips Medical Systems Achieva 3 T) and a 1.5 Tesla machine (MRC21271 SIEMENS Sonata). Axial plane images were obtained in T1, T2, FLAIR (Fluid Attenuation Inversion Recovery), DWI (Diffusion-Weighted Imaging) and SWI (Susceptibility Weighted Imaging) sequences. Carotid magnetic resonance angiography (MRA) was performed using the 3D-TOF sequence. Individuals were examined according to the specific protocol of the hospital's imaging centre. Two neuroradiologists blinded to the clinical data reviewed the exams and the final report was obtained after consensus.

The following parameters were assessed in the brain MRI scans of patients who underwent the imaging protocol: territorial infarction, multiple or single acute microembolism, localized cortical infarction, border infarction, lacunar infarction, ischaemic lesions in any territory, white matter hyperintensity, micro and macro haemorrhages and arterial stenosis ≥50% of the cervical carotid artery in MRA. These parameters have been previously described in the literature [21, 22]. White matter hyperintensity (WMH) was classified according to the Fazekas scale [23].

### Statistical analysis

The Kolmogorov–Smirnov test was used to evaluate normality. Categorical variables were presented as frequency and percentage, while continuous variables were presented as mean and standard deviation. Categorical variables were compared using the chi-square test. Parametric qualitative variables were compared using ANOVA, followed by Bonferroni post-test. Non-parametric qualitative variables were compared using the Kruskal-Wallis test and the Mann–Whitney U tests. Statistical analysis was performed using SPSS software version 20.0 (Chicago, IL, USA). A *P*-value < 0.05 was considered significant.

## Results

### Clinical and epidemiological data

Demographic and laboratory data of the patients’ groups are compared in Table 1. The cohort comprised predominantly female individuals, constituting 85 (90.4%) of the study population, with a subset of 26 (27.6%) identified as Caucasian. Educational levels were higher in the control group compared with the disease-specific groups (*P* < 0.00). Duration of the disease since APS diagnosis did not differ significantly between the PAPS and SAPS groups, with means reported at 8.5 ± 7 years and 11.7 ± 7 years, respectively. Furthermore, the mean duration from SLE diagnosis was comparable between the SAPS (24 ± 9 years) and SLE cohorts (26 ± 9.2 years).

**Table 1.** Demographic and laboratory data

	PAPS (*n* = 22)	SAPS (*n* = 24)	SLE (*n* = 27)	Control (*n* = 21)	*P*-value
Age, mean (s.d.), years	44.6 (10.2)	43.6 (11.6)	43.7 (12)	43.2 (12.4)	0.982
Gender					
Male	3(13.6%)	2(8.3%)	2(7.4%)	2(9.5%)	0.894
Female	19(86.4%)	22(91.7%)	25(92.6)	19(90.5%)	
Ethnicity					
White	3(13.6%)	9(37.5%)	5(18.5%)	9(42.8%)	0.085
Non-White	19(86.4%)	15(62.5%)	22(81.5%)	12(57.2%)	
School, mean (s.d.), years	11.1 (3.8)	12.7 (3.6)	10.65 (4.3)	16.95 (3.8)*	<0.001
Disease duration (APS), mean (s.d.), years	8.5 (7)	11.7 (7)	–	–	0.126
Disease duration (SLE), mean (s.d.), years		24 (9)	26 (9.2)	–	0.753
Laboratory profile					
Haemoglobin, mean (s.d.)	12.3 (1.63)	12.3 (2.1)	12.3 (1.7)	–	1.0
Hematocrit, mean (s.d.)	37.6 (4.4)	35.7 (7.8)	37.7 (5.2)	–	0.452
Prothrombin time, mean (s.d.)	2.23 (0.9)	2.27 (0.8)	1.03 (0.2)*	–	<0.001

PAPS: primary antiphospholipid syndrome; SAPS: secondary antiphospholipid syndrome.

*The group presented a *P*-value < 0.01, indicating a significant difference compared to the other groups (*P* < 0.05).

There were no observed differences in clinical manifestations that meet the criteria for antiphospholipid syndrome between the PAPS and the SAPS groups, nor were there differences in the immunological profiles (Table 2).

**Table 2.** Clinical data of APS, SAPS and SLE patients

	PAPS (*n* = 22)	SAPS (*n* = 24)	SLE(*n* = 27)	*P*-value
Cerebrovascular manifestations				
Stroke	9(40.9%)	9(37.5%)	1(3.7%)*	0.004
Transient ischaemic attack	2(9.1%)	0(0%)	1(3.7%)	0.297
Cerebral venous thrombosis	2(9.1%)	1(4.2%)	0(0%)	0.280
Cardiovascular risk factors				
Arterial hypertension	10(45.5%)	8(33.3%)	14(51.9%)	0.406
Diabetes	4(18.2%)	1(4.2%)	1(3.7%)	0.126
Dyslipidaemia	9(40.9%)	5(20.8%)	11(40.7%)	0.240
Renal chronic disease	2(9.1%)	1(4.3%)	4(14.8%)	0.434
Obesity	6(27.3%)	9(37.5%)	9(33.3%)	0.760
Myocardial infarction	1(4.5%)	0(0%)	0(0%)	0.309
Smoking	2(9.1%)	1(4.2%)	1(3.7%)	0.671
Carotid Stenosis (>50%)	0(0%)	0(0%)	1(3.7%)	0.422
Cardiac arrhythmia	1(4.5%)	0(0%)	0(0%)	0.309
Medications				
Immunosuppressant	3(13.6%)*	14(58.3%)	21 (77.8%)	<0.001
Steroids	1(4.3%)*	8(34.8%)	14(60.9%)	<0.001
Aspirin	4(18.2%)	6(25%)	6(22.2%)	0.855
Anticoagulant	18(81.8%)	19(752%)	1(3.7%)*	<0.001
Statins	6(27.3%)	7(29.2%)	6(22.2%)	0.842

PAPS: primary antiphospholipid syndrome; SAPS: secondary antiphospholipid syndrome.

*The group presented a *P*-value < 0.01, indicating a significant difference compared to the other groups (*P* < 0.05).

Previous stroke was the most common cerebrovascular manifestation and was more common in patients with PAPS and SAPS than in patients with SLE (*P* =* *0.004). The frequency of cardiovascular risk factors was similar between patients with PAPS, SAPS and SLE. Hypertension, dyslipidaemia and obesity were the predominant risk factors observed in these patient groups. Most patients with APS were using antithrombotic therapy. Anticoagulation was more frequent in the PAPS and APS/SLE groups than in the SLE group (Table 3).

**Table 3.** Transcranial Doppler findings between groups

	PAPS	SAPS	SLE	CON	*P*-value
	(*n* = 22)	(*n* = 24)	(*n* = 27)	(*n* = 21)	
Spontaneous MES	0 (0%)	1(4.5%)	4(14.8%)*	0(0%)	0.037
Intracranial stenosis	2(9.1%)	3(12.5%)	2(7.4%)	0(0%)	0.446
RLS positive	14(63.6%)	14(63.6%)	15(55.6%)	8(38.1%)	0.289
RLS classification					
Grade I	8(36.4%)	9(40.9%)	11(42.3%)	8(36.4%)	
Grade II	7(31.8%)	9(40.9%)	9(34.6%)	1(5%)	0.264
Grade III	6(27.3%)	3(13.6%)	3(11.5%)	6(30%)	
Grade 4 IV	1(4.5%)	1(4.5%)	3(11.5%)	1(5%)	
Breath—Holding Index (BHI)	1.49 ± 0.40	1,23 ± 0.90	1.56 ± 0.50	1.48 ± 0.32	0.06

PAPS: primary antiphospholipid syndrome; SAPS: secondary antiphospholipid syndrome; MES: microembolic signal; RLS: right–left shunt.

*The group presented a *P*-value of 0.037, indicating a significant difference compared to the other groups (*P* < 0.05).

### TCD findings

Patients with APS (either PAPS or SAPS) had a higher frequency of RLS when compared with healthy volunteers (63.6% vs 38.1%, p.0.05). There was no difference when comparing the four groups. The degree of RLS did not differ among the groups (Table 4). The frequency of intracranial stenosis was similar among the groups. Although a higher proportion of patients with APS (primary or secondary) presented with more intracranial stenosis than healthy volunteers, the difference was not statistically significant (10.9% vs 0, *P* = 0.11). MES were more frequently found in patients with SLE when compared with the other groups (SLE: 17.4%, SAPS: 4.3%, MES was not found in patients with PAPS nor healthy controls, *P* = 0.03). The disparity in MES occurrence between patients receiving anticoagulation therapy (2.9% vs 12.1%, p.0.14). VMR measured by BHI values had a trend to be lower in patients with SAPS than in control subjects and patients with either PAPS or SLE (PAPS 1.23 ± 0.9, SAPS 1.49 ± 0.40, SLE 1.56 ± 0.50, controls 1.48 ± 0.32, *P* = 0.06). There were no significant differences in MFV nor PI among the groups in all arteries evaluated. There was no association between the history of cerebrovascular disease and the presence of RLS, MES, intracranial stenosis, or altered cerebral vasomotor reactivity. The detailed TCD results of our series were previously published [16].

**Table 4.** Neuroimaging findings

Neuroimaging findings	PAPS	SAPS	SLE	*P*-value
	(*n* = 20)	(*n* = 19)	(*n* = 19)	
Ischaemic lesion in any territory	10 (50%)	5 (26.3%)	5 (26.3%)	0.177
Acute multiple or single microembolism	0 (0%)	0(0%)	0 (0%)	–
Localized infarction	6 (30%)	2 (10.5%)	2 (10.5%)	0.175
Lacunar infarction	6 (30%)	0 (0 %)	2 (10.5%)	0.022
Lesions larger than lacunar infarcts	1 (5%)	1 (5.3%)	0 (0%)	0.603
White matter hyperintensity				
Deep white substance	12 (60%)	12 (63.2%)	13 (68.4%)	0.859
Fazekas grade 0	0/12 (0%)	0/12 (0%)	0 (0%)	
Fazekas grade I	9/12 (75%)	7/12 (58.3%)	9/13 (69.2%)	0.777
Fazekas grade II	2/12 (16.7%)	4/12 (33.3%)	2/13 (15.4%)	
Fazekas grade III	1/12 (8.3%)	1/12(8.3%)	2/13 (15.4%)	
Periventricular white substance				
Fazekas grade 0	0/12 (0%)	2/12 (16.7%)	2/13 (15.4%)	0.495
Fazekas grade I	6/12 (50%)	8/12 (66.7%)	8/13 (61.5%)	
Fazekas grade II	5/12 (41.7%)	2/12 (16.7%)	2/13 (15.4%)	
Fazekas grade III	1/12 (8.3%)	0/12 (0%)	1/13 (7.7%)	
Microhaemorrhage	1 (5%)	0 (0%)	0 (0%)	0.380
Macrohaemorrhage	3 (15%)	1 (5.3%)	0 (0%)	0.171

PAPS: primary antiphospholipid syndrome; SAPS: secondary antiphospholipid syndrome; MES: microembolic signal; RLS: right–left shunt.

### MRI findings

Among the 58 patients who underwent neuroimaging protocol, 54 patients were scanned using a 3.0 Tesla magnetic field MRI machine and four patients using a 1.5 Tesla machine. The reasons for 15 patients not undergoing brain MRI were loss of follow-up/contact (*n* = 5), failure to attend the exam (*n* = 3), claustrophobia (*n* = 3) and other causes (refusal to undergo the exam = 2, use of metallic orthodontic appliance = 1, eyebrow hyperpigmentation procedure = 1). (Supplementary Table S1, available at *Rheumatology Advances in Practice* online).

The most common brain MRI findings were white matter disease in T2/FLAIR and ischaemic lesions (Table 5). WMH was present in 60% of patients with PAPS, 63.2% with SAPS and 68.4% with SLE. Comparative analysis revealed no significant variance in the Fazekas scale scoring for WMH across the studied groups. Ischaemic lesions occurred in 50% of patients with PAPS, 26.3% with SAPS and 26.3% with SLE. There was no difference between the groups regarding the frequency of ischaemic lesions in any territory, territorial infarction, localized infarction, acute multiple or single microembolism, WMH, larger-than-lacunar infarcts, microhaemorrhages, macrohaemorrhages and carotid stenosis. Only the frequency of lacunar infarction was significantly different among the three groups (PAPS: 30%, SAPS: 0% and SLE: 10.5%, *P* = 0.022). This finding was more frequent in patients with PAPS than patients with SAPS (*P* = 0.009), but there was no difference when comparing the other groups.

**Table 5.** Frequency of ischaemic alterations on brain MRI according to DTC finding

Neuroimaging findings	MES			Intracranial stenosis			Shunt right-left		
	Present	Absent	*P*-value	Present	Absent	*P*-value	Present	Absent	*P*-value
	(*n* = 3)	(*n* = 55)		(*n* = 5)	(*n* = 53)		(*n* = 35)	(*n* = 21)	
Ischaemic lesion in any territory	1 (33.3%)	18 (32.7%)	1.0	3 (60%)	17 (32.1%)	0.209	13 (37.1%)	7 (33.3%)	0.773
Acute multiple or single microembolism	0 (0%)	6 (10.9%)	0.542	1 (40%)	4 (7.5%)	0.023	3 (8.6%)	3 (14.3%)	0.503
Localized infarction	1 (33.3%)	9 (18.1%)	0.460	1 (20%)	9 (17%)	0.864	6 (17.1%)	4 (19%)	0.857
Lacunar infarction	0 (0%)	8 (14.5%)	0.472	2 (40%)	6 (11.3%)	0.075	9 (25.7%)	3 (14.3%)	1.00
Lesions larger than lacunar infarcts	0 (0%)	2 (3.6%)	0.734	1 (20%)	1 (1.9%)	0.034	2 (5.7%)	0 (0%)	0.265
Ischaemic lesion in any territory	0 (0%)	2 (3.6%)	0.734	0 (0%)	2 (3.8%)	0.658	1 (2.9%)	1 (4.8%)	0.710

PAPS: primary antiphospholipid syndrome; SAPS: secondary antiphospholipid syndrome; MES: microembolic signal; RLS: right–left shunt.

The presence of RLS, the degree of RLS, or alterations in vasoreactivity were not associated with the frequency of ischaemic lesions in any territory, territorial infarction, localized infarction, lacunar infarction, or lesions larger than lacunar infarcts. The occurrence of MES also was not associated with ischaemic lesions on brain MRI (Supplementary Table S1, available at *Rheumatology Advances in Practice* online).

Patients with intracranial stenosis detected by TCD had a higher frequency of territorial infarction (40% vs 7.5%, *P* = 0.02), lacunar infarction (40% vs 11.3%, *P* = 0.075) and border zone infarction (20% vs 1.9%, *P* = 0.034). There was no association with other assessed neuroimaging parameters (Supplementary Table S1, available at *Rheumatology Advances in Practice* online).

## Discussion

In our study, APS and SLE patients with intracranial stenosis detected by TCD had a higher frequency of cerebral infarctions (territorial, lacunar and border zone infarcts) compared with APS and SLE patients who did not have intracranial stenosis. Additionally, SLE patients had a more significant number of microembolic signals (MES) than patients with PAPS, SAPS and the healthy control group. We also found that APS patients were more likely to have RLSs than individuals in the control group.

The prevalence of intracranial stenosis in our series was higher than previously described and not different between groups [24–27]. Several potential explanations for the elevated incidence of intracranial stenosis in our population can be considered, including a heightened prevalence of traditional risk factors associated with intracranial atherosclerotic disease, such as hypertension, diabetes, dyslipidaemia and obesity, within our study sample. Additionally, our study population had an older mean age compared with previous studies and it consisted primarily of non-Caucasian individuals (intracranial atherosclerotic disease is more prevalent among Black and Asian populations). It is important to note that there may be several unexamined variables of significance and vascular findings are often multifactorial, encompassing factors such as disease severity and suboptimal anticoagulation management. Intracranial stenosis is an important risk factor for acute ischaemic cerebrovascular events (stroke and TIA), responsible for approximately 10–15% of cases in the general population [28]. In patients with PAPS, it may be due to antiphospholipid antibody-induced vasculopathy or intracranial atherosclerosis. Despite representing a potential mechanism for acute ischaemic cerebrovascular events in patients with PAPS, the actual prevalence of intracranial stenosis and its impact on the occurrence of acute ischaemic cerebrovascular events in this group of patients are still unknown [21].

The main neuroimaging findings in patients with APS were the presence of ischaemic lesions and white matter hyperintensity, consistent with previous studies. In the literature, the frequency of these abnormalities varies depending on the characteristics of the studied population and appears to be more prevalent in patients with neurological symptoms [10, 29, 29]. Previous studies have not directly compared the three groups regarding neuroimaging findings and only one study has compared PAPS with SAPS patients [10]. In this study, the frequency of radiological abnormalities was similar between the groups, but PAPS patients had a higher incidence of infarctions involving larger vascular territories. However, this study had limitations, including using computed tomography instead of MRI for some patients and lacking detailed information on antiphospholipid antibody titres for APS diagnosis.

Previous studies found an association between APS and brain MRI abnormalities in SLE patients [30, 31]. A study with studied 256 SLE patients (45 with PAPS and 211 without PAPS) found that abnormal brain MRI findings were more common in those with PAPS [30]. This included lacunar, cortical, or border zone ischaemic lesions and arterial or basal ganglia stenosis. The association was independent of age. Another study with 325 SLE patients showed that antiphospholipid antibodies, regardless of APS diagnosis, increased the risk of ischaemic brain lesions [31]. Factors associated with these lesions were positive lupus anticoagulant, systemic hypertension and accumulated disease damage.

Few studies have explored the correlation between brain MRI data and TCD findings in patients with PAPS and even fewer have included patients with SLE. Among these, one study analysed 55 SLE patients (including 11 with associated PAPS) and investigated the relationship between MES and cerebral infarction on MRI, revealing a positive correlation. Similarly, another study with 23 SLE patients unveiled an association between silent cerebral embolism, the occurrence of High Signal Brightness on brain MRI and neuropsychiatric symptoms. Nonetheless, neither of these studies [32, 33] examined a comprehensive array of sonographic and neuroimaging parameters as we did in our series.

In our cohort, there was no association between the presence of RLS, shunt grade, or vasoreactivity changes and neuroimaging findings. The occurrence of MES was not associated with a higher frequency of ischaemic changes on brain MRI and was not a risk marker for cerebrovascular events in the studied population. However, the assessment of this association was possibly hindered by the fact that many patients with a history of previous thrombotic events were on oral anticoagulation.

Despite the significance of these findings, our study has some limitations that could impact interpretation. The small sample size, with 22% of patients not undergoing brain MRI, may limit the generalizability of our results. Furthermore, the images were obtained using two distinct machines, with most patients (54 out of a total of 58) being subjected to the 3 T protocol. Lastly, some clinical data were gathered retrospectively from medical records, introducing the possibility of information bias. Nevertheless, it is important to highlight that, despite these constraints, our study rigorously employed statistical methodologies to ensure the robustness and validity of our findings. By carefully designing the study and selecting appropriate statistical analyses, we aimed to mitigate the impact of the smaller sample size and the use of different imaging equipment on our results. This approach allowed us to draw meaningful conclusions from the data, contributing valuable insights into the complex interplay between sonographic findings and neuroimaging abnormalities in patients with APS. Our study included the largest number of patients with PAPS monitored with TCD and was the first to correlate sonographic findings with clinical and neuroimaging examinations in this population.

## Conclusion

Our study presents novel findings regarding the association between intracranial stenosis and ischaemic injury in neuroimaging studies among patients with SLE and APS, thereby proposing a possible aetiological mechanism for cerebrovascular ischaemic events in this population. Notably, the frequency of brain MRI abnormalities was comparable among patients with SLE, PAPS and SAPS, except for lacunar infarction. Our findings highlight the need to evaluate intracranial vessels in patients with SLE or APS who present with cerebrovascular ischaemic events. Brain Magnetic Resonance Angiography is often overlooked in such patients, as cerebrovascular ischaemic events are commonly attributed to the pro-thrombotic state. Our findings contribute to the knowledge of cerebrovascular disease mechanisms in patients with autoimmune diseases, especially PAPS and SLE and emphasize the importance of jointly evaluating clinical, sonographic and neuroimaging parameters to better understand these conditions. Further studies with a larger population and including patients not under anticoagulant therapy are important to deepen the understanding of our results.

## Supplementary Material

rkae060_Supplementary_Data

## Data Availability

The data supporting this study's findings are included within the article and its Supplementary Material, available at *Rheumatology Advances in Practice* online.
