# Supplementary material for: Cerebrovascular disease in patients with antiphospholipid antibody syndrome: a transcranial Doppler and magnetic resonance imaging study
Source: Rheumatol Adv Pract. 2024 May 7;8(2):rkae060. doi: 10.1093/rap/rkae060 (PMC11127108; doi:10.1093/rap/rkae060)
Supplement: rkae060_Supplementary_Data [file rkae060_supplementary_data.docx]

**Supplementary Table S1: Frequency of ischemic alterations on brain MRI according DTC finding.**



| Neuroimaging findings | MES | | | Intracranial Stenosis | | | Shunt right-left | | |
| --- | --- | --- | --- | --- | --- | --- | --- | --- | --- |
|  | **Present**  (N = 3) | **Absent**  (N=55) | **p-value** | **Present**  (N = 5) | **Absent**  (N= 53) | **p-value** | **Present**  (N = 35) | **Absent**  (N =21) | **p-value** |
| Ischemic lesion in any territory | 1 (33.3%) | 18 (32.7%) | 1.0 | 3 (60%) | 17 (32.1%) | 0.209 | 13 (37.1%) | 7 (33.3%) | 0.773 |
| Acute multiple or single microembolism | 0 (0%) | 6 (10.9%) | 0.542 | 1 (40%) | 4 (7.5%) | 0.023 | 3 (8.6%) | 3 (14.3%) | 0.503 |
| Localized infarction | 1 (33.3%) | 9 (18.1%) | 0.460 | 1 (20%) | 9 (17%) | 0.864 | 6 (17.1%) | 4 (19%) | 0.857 |
| Lacunar infarction | 0 (0%) | 8 (14.5%) | 0.472 | 2 (40%) | 6 (11.3%) | 0.075 | 9 (25.7%) | 3 (14.3%) | 1.00 |
| Lesions larger than lacunar infarcts | 0 (0%) | 2 (3.6%) | 0.734 | 1 (20%) | 1 (1.9%) | 0.034 | 2 (5.7%) | 0 (0%) | 0.265 |
| Ischemic lesion in any territory | 0 (0%) | 2 (3.6%) | 0.734 | 0 (0%) | 2 (3.8%) | 0.658 | 1 (2.9%) | 1 (4.8%) | 0.710 |

PAPS (Primary Antiphospholipid Syndrome); SAPS (Secondary Antiphospholipid Syndrome); SLE (Systemic lupus erythematous); MES (Microembolic Signal); RLS (Right – Left Shunt).
